# MK-2206 sensitizes BRCA-deficient epithelial ovarian adenocarcinoma to cisplatin and olaparib

**DOI:** 10.1186/s12885-016-2598-1

**Published:** 2016-07-27

**Authors:** Margaret E. Whicker, Z. Ping Lin, Ruth Hanna, Alan C. Sartorelli, Elena S. Ratner

**Affiliations:** 1 Department of Obstetrics, Gynecology, and Reproductive Sciences, Yale University School of Medicine, 333 Cedar Street, New Haven, CT USA; 2 Section of Gynecologic Oncology, Department of Obstetrics, Gynecology, and Reproductive Sciences, Yale University School of Medicine, 333 Cedar Street, New Haven, CT USA; 3 Department of Pharmacology, Yale University School of Medicine, 333 Cedar Street, New Haven, CT USA

**Keywords:** Epithelial ovarian cancer, PARP inhibitors, MK-2206, AKT inhibitors, BRCA

## Abstract

**Background:**

Platinum resistance is a major obstacle in the treatment of epithelial ovarian cancer (EOC). Activation of the AKT pathway promotes platinum resistance while inhibition of AKT sensitizes chemoresistant cells. Patients with BRCA mutant EOC, and thus a defect in the homologous recombination (HR) repair pathway, demonstrate greater clinical response to platinum and olaparib therapy than patients with BRCA wild-type EOC. MK-2206, an allosteric inhibitor of AKT phosphorylation, sensitizes a variety of cell types to various anticancer agents and is currently undergoing phase II trials as monotherapy for platinum-resistant ovarian, fallopian tube, and peritoneal cancer. This study examines the differential effects of AKT inhibition with cisplatin and olaparib therapy in BRCA1/2-deficient versus wild-type EOC.

**Methods:**

PEO1, a chemosensitive BRCA2-mutant serous ovarian adenocarcinoma, and PEO4, a reverted BRCA2-proficient line from the same patient after the development of chemotherapeutic resistance, were primarily used for the study. In PEO1, MK-2206 demonstrated moderate to strong synergism with cisplatin and olaparib at all doses, while demonstrating antagonism at all doses in PEO4.

**Results:**

Baseline phospho-AKT activity in untreated cells was upregulated in both BRCA1- and 2-deficient cell lines. MK-2206 prevented cisplatin- and olaparib-induced AKT activation in the BRCA2-deficient PEO1 cells. We propose that BRCA-deficient EOC cells upregulate baseline AKT activity to enhance survival in the absence of HR. Higher AKT activity is also required to withstand cytotoxic agent-induced DNA damage, leading to strong synergism between MK-2206 and cisplatin or olaparib therapy in BRCA-deficient cells.

**Conclusions:**

MK-2206 shows promise as a chemosensitization agent in BRCA-deficient EOC and merits clinical investigation in this patient population.

## Background

Epithelial ovarian cancer (EOC) is the leading cause of death among women with pelvic reproductive organ cancer in the United States, with over 22,280 cases diagnosed and 15,500 deaths each year [1]. Despite the introduction of new approaches to therapy, the high mortality rate of EOC has remained largely static for many years, with a 5-year overall survival rate of only 44.1 % in patients diagnosed between 2003 and 2009 [2]. Based on multiple phase III studies, the current standard of care in the treatment of EOC is maximal surgical cytoreduction followed by platinum-based chemotherapy, most commonly carboplatin, in combination with paclitaxel [3–5]. On the platinum-taxane regimen, up to 70-80 % percent of patients will enter remission [6]. However, despite this often excellent response to primary therapy, approximately 65 % of patients will ultimately experience disease progression and require further treatment [7]. At all stages of disease, progression-free survival and overall survival depend greatly on the tumor sensitivity to platinum chemotherapy. For patients who become resistant to platinum therapy, response to other cytotoxic chemotherapeutic regimens is low, with response rates of only 6-30 % [8].

Given the direct association between platinum resistance and disease prognosis, the underlying mechanisms resulting in platinum resistance are a focus of substantial investigation. Various molecular mechanisms of platinum resistance have been postulated, including alterations in the AKT/mTOR and homologous recombination (HR) repair pathways [9–11]. AKT, a serine/threonine kinase family that is activated in a PI-3-K-dependent manner, is involved in pathways regulating cell growth and protein synthesis, entry into the cell cycle, and cellular survival [12]. Activation of the AKT pathway has been shown to promote a platinum-resistant phenotype, whereas inhibition of AKT sensitizes chemoresistant cells to cisplatin-induced apoptosis [13]. Activation of AKT also prevents cisplatin-induced phosphorylation and activation of p53, required for the apoptotic response to cisplatin treatment [14]. In addition, AKT2 is activated above baseline in approximately 40 % of primary high-grade ovarian cancers and transcriptionally amplified in a further 12 % [15, 16]. Inhibition of AKT1 and AKT2 has been demonstrated to selectively sensitize tumor cells to apoptotic stimuli without commensurate effects on normal cells [17]. MK-2206 is an orally active allosteric inhibitor of AKT that prevents AKT1 and AKT2 phosphorylation at both the Thr308 and Ser473 sites, and also prevents AKT-mediated phosphorylation of downstream targets [18, 19]. It has been previously shown to sensitize multiple human tumor cell lines to a variety of anticancer agents [20], and is currently in phase II trials as a single agent therapy for patients with recurrent platinum-resistant ovarian, fallopian tube, and peritoneal carcinoma (NCT01283035).

Other cellular responses to platinum-induced DNA damage may also be involved in platinum resistance. HR is a major mechanism for the repair of DNA double-strand breaks (DSBs) [21]. Integral to this process are the well-known tumor suppressor genes BRCA1 and BRCA2. EOC with BRCA1 or BRCA2 mutations has compromised HR activity and has long been known to exhibit increased sensitivity to platinum drugs [22–25]. Additionally, the restoration of BRCA1/2 function in initially BRCA1/2-deficient EOC has been linked to the development of platinum resistance. The secondary restoration of BRCA1 function has been shown in a number of originally mutant EOC cell lines after resistance had developed to cisplatin [26]. Defective HR repair also renders BRCA-deficient cells susceptible to poly (ADP ribose) polymerase (PARP) inhibitors, which compromise base excision repair (BER), a complementary DNA repair pathway [27]. Olaparib (AZD-2281, KU59436) is an oral third-generation PARP-1 inhibitor that has demonstrated substantial antitumor activity in BRCA-deficient EOC. Notably, both pharmacological inhibition and siRNA-knockdown of PARP-1 have been demonstrated to induce activation of the anti-apoptotic AKT pathway and promote resistance to paclitaxel, raising concern for the need for a specific AKT inhibitor to circumvent this drug-induced drug resistance mechanism [28].

A number of studies have elucidated an intricate relationship between BRCA proteins and AKT activity. BRCA1 has been implicated as a negative regulator of AKT, targeting phosphorylated AKT for ubiquitination and degradation in mammary tumors [29]. In addition, the absence of BRCA2 has been implicated in increased AKT signaling in prostate cancer, leading to increased cell proliferation [30]. Conversely, AKT has also been shown to antagonize the activity of BRCA1. In wild-type sporadic breast cancer lines, AKT1 has been shown to promote cytoplasmic retention of BRCA1 and Rad51. As both BRCA1 and Rad51 require nuclear localization to participate in HR repair, this AKT1 activity represses HR activity and creates a functional phenotype similar to a BRCA1 mutant, commonly referred to as “BRCAness” [31, 32].

To date, no studies have elucidated the differential effects of AKT inhibition on BRCA mutant versus wild-type cell lines. Given the mutually antagonistic relationship between AKT and BRCA1, and the repressive action of AKT on HR repair, we hypothesized that BRCA mutants might demonstrate higher levels of AKT activity despite demonstrating increased susceptibility to DNA-damaging agents. Additionally, the inhibition of AKT in BRCA mutants might render cells more sensitive to DNA-damaging agents, such as cisplatin. In this study, we examined the effect of AKT inhibition with MK-2206 on the sensitivity of paired BRCA-proficient and –deficient EOC cell lines to cisplatin and olaparib treatment.

## Methods

### Chemicals

Cisplatin was obtained from Calbiochem/EMD Millipore (Billerica, MA, USA). Olaparib (AZD-2281), a selective PARP1/PARP2 inhibitor, and MK-2206, a selective inhibitor of AKT1/AKT2/AKT3, were obtained from Selleck Chemicals (Houston, TX, USA).

### Experimental cell lines

The human ovarian adenocarcinoma cell lines PEO1 and PEO4 were generously provided by Dr. Peter Glazer (Yale University School of Medicine, New Haven, CT, USA). PEO1 is a chemosensitive BRCA2-mutant poorly differentiated serous ovarian adenocarcinoma derived from malignant ascites of a patient with a BRCA2 germline mutation 22 months after initial treatment with cisplatin, 5-fluorouracil and chlorambucil. The patient was subsequently retreated with platinum based therapy and had a further 10 month progression free interval, indicating the platinum sensitivity of the disease at the time of PEO1 retrieval. PEO4, a reverted BRCA2-proficient line was derived from the same patient after the development of chemotherapeutic resistance (the patient subsequently received high dose platinum therapy with rapid progression) [33, 34]. Cells were maintained in logarithmic growth in DMEM media with 10 % FBS and penicillin/streptomycin antibiotics. To evaluate the potential applicability of our findings to BRCA1 deficient EOC, preliminary studies to determine AKT activity and MK-2206 sensitivity were performed in the BRCA1 wild-type human ovarian adenocarcinoma line SK-OV-3 (ATCC; Manassas, VA, USA). Non-targeted siRNA control (NTC) and BRCA1-knockdown (BRCA1-kd) SK-OV-3 cell lines were established in our lab as described previously [35, 36]. Cells were maintained in logarithmic growth in McCoy’s 5A media, supplemented with 10 % fetal bovine serum (FBS) and penicillin-streptomycin antibiotics. All cell lines used in this study are commercially available and Human Investigation Committee approval was not required.

### Clonogenic assays

SK-OV-3 NTC and BRCA1-kd cells were seeded in triplicate at various densities in 6-well plates. After 24 h of incubation, cells were treated continuously with single drugs or combinations of cisplatin, olaparib, and MK-2206. Plates were then incubated for 14 days, at which point colonies were fixed and stained with crystal violet/methanol (0.5 % crystal violet, 50 % methanol) solution. All clonogenic assays used triplicate cultures of each cell line, repeated three times. Colony counts were obtained with an automated Bio-Rad GelDoc imaging system and QuantityOne analysis software (Bio-Rad; Hercules, CA, USA).

### Cytotoxic assays

PEO1 and PEO4 cells were seeded in 96-well plates at a density of 5,000 cells/well. After 24 h of incubation, cells were treated with single drugs or combinations of cisplatin, olaparib, and MK-2206 in a fixed ratio of 1:1. After 72 h of continuous incubation, wells were treated with CellTiter 96 AQueous One Solution Cell Proliferation Assay Reagent (Promega; Madison, WI, USA). All cytotoxic assays used triplicate cultures of each cell line, repeated three times. Cells were incubated for 4 h before absorbances were read at 490 nm with a colorimetric microplate reader. Data are means ± standard deviation. Student T-tests were performed to compare survival across cell lines.

### Drug interaction analysis

To identify synergistic/antagonistic drug interactions, Combination Indices (CI) were determined with CalcuSyn software (Biosoft; Cambridge, UK) using the Chou-Talalay method [37]. A CI > 1.00 indicates an antagonistic interaction between two drugs, and a CI < 1.00 indicates a synergistic interaction. A CI near 1.00 represents near-additive effects and minimal drug-drug interaction.

### Western blot analysis

Cells were plated in 60-mm plates and incubated for 24 h prior to drug treatment, and then were incubated for an additional 24 h with continuous drug exposure until cells had reached 80 % confluence. For time course experiments, the cells were incubated with continuous drug exposure and lysed immediately following treatment (Oh), and subsequently at 3, 6, 12, and 24 h. Cells were lysed and protein concentrations were determined by the Bio-Rad DC protein assay according to the manufacturer's instructions. Forty micrograms of protein were resolved by electrophoresis in a 4–20 % polyacrylamide Mini-PROTEAN TGX precast gel (BioRad) and transferred onto a nitrocellulose membrane. The membrane was blocked with 5 % milk in TBST (Tris-buffered saline with 0.05 % Tween-20) for 1 h at room temperature and incubated with primary antibody in the blocking solution at 4 °C overnight. The membrane was subsequently washed with TBST, incubated with a horseradish peroxidase-conjugated secondary antibody in blocking solution at room temperature for 1 h, and washed again. The target protein was visualized by an enhanced chemiluminescence reagent (Denville Scientific; South Plainfield, NJ, USA). Images were obtained with the Syngene ChemDoc imaging system (Syngene; Frederick, MD, USA). HSC-70 protein, a constitutively expressed member of the 70-kDa heat shock protein family, was also used as a secondary loading control in the event of uneven total AKT expression via the procedure described above [38, 39]. Anti-phospho-AKT (Ser473; 193H12) (Thr308; 244 F9), anti-AKT (40D4), anti-phospho-S6 ribosomal protein (Ser235/236; 2 F9), anti-S6 ribosomal protein (54D2), and anti-PARP antibodies were purchased from Cell Signaling Technology (Beverly, MA, USA). Anti-HSC-70 (B-6) antibodies were purchased from Santa Cruz Biotech (Dallas, TX, USA). Individual western blotting analyses were performed at least three times with separately prepared lysates and a representative blot was chosen for display. Band intensity was quantified with ImageJ software (National Institute of Health, Bethesda, MD, USA).

### Apoptosis assay

Cells were seeded in 6-well plates, and treated with drug combinations of cisplatin or olaparib and MK-2206 after 24 h of incubation. After 72 h of continuous drug exposure, cells were lysed (PBS, 1 % NP40, 0.1 % SDS). Cell lysate (5 μl) was incubated with Caspase-Glo 3/7 Assay reagent (Promega) at room temperature for 1 h and subsequently luminescence was measured with a TD-20/20 luminometer (Turner Designs/Promega). Total protein concentration of cell lysates was determined as described above. Caspase 3/7 activity was normalized to total protein concentration and expressed as relative luminescence units (RLU) per μg protein.

## Results

### BRCA mutant cell lines demonstrate higher levels of baseline AKT activity

**Fig. 1:**
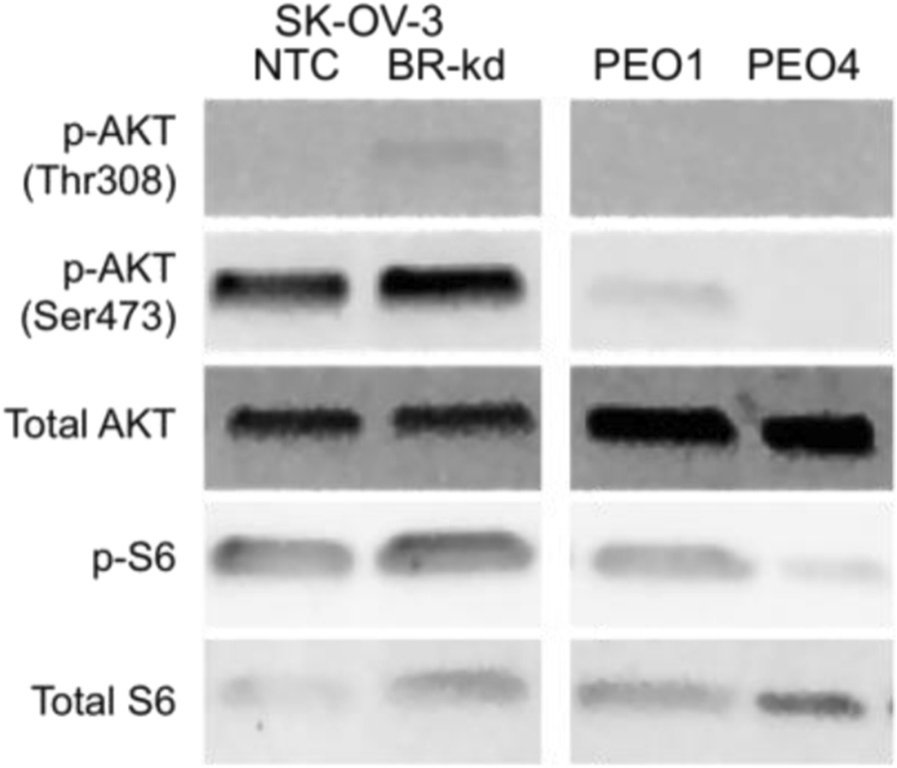
BRCA mutant cell lines demonstrate higher levels of baseline AKT activity. PEO1, PEO4, and SK-OV-3 NTC and BRCA1-kd cells were untreated and levels of phosphorylated and total AKT and S6 proteins were assessed by western blot analysis

In both the SK-OV-3 and PEO paired cell lines, untreated cells showed similar baseline levels of total AKT. However, both SK-OV-3 BRCA1-kd and PEO1, the BRCA1-deficient and BRCA2 mutant cell lines, respectively, showed higher levels of AKT phosphorylation at the Ser473 site than in their BRCA-wild-type counterparts (Fig. 1). SK-OV-3 BRCA1-kd also showed higher levels of AKT phosphorylation at Thr308. In all western blotting experiments, PEO1 and PEO4 did not demonstrate any detectable phosphorylation at the Thr308 site and this is not shown going forward. Ribosomal S6 is a downstream target of phospho-AKT (p-AKT) and its phosphorylation status is useful as an indicator of activation of the AKT pathway [40]. The BRCA-deficient PEO1 displayed higher levels of phosphorylated S6 than the BRCA2-proficient PEO4.

### BRCA mutant cell lines are more susceptible to cisplatin and olaparib treatment

**Fig. 2:**
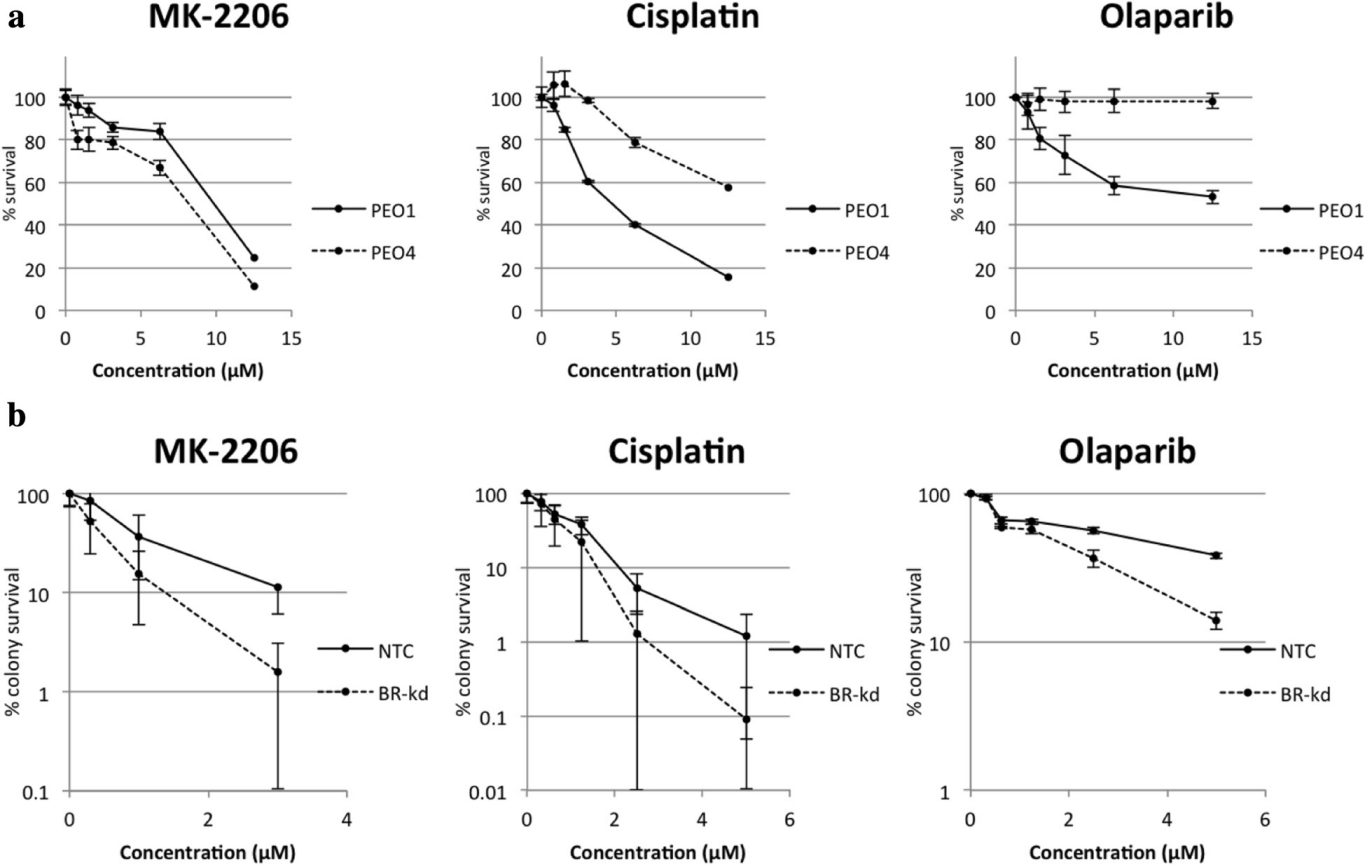
BRCA mutant cell lines are more susceptible to cisplatin and olaparib treatment. **a** Cytotoxicity Assay: PEO1 and PEO4 cells were treated with various concentrations of MK-2206, cisplatin, and olaparib. After 72 h, MTS solution was added and absorbance read at 490 nm after 4 h. Data are means ± SD. Survival is significantly different (*p* < 0.001) at all points with the exception of the 0.78 μM concentration of olaparib (*p* = 0.27). **b** Clonogenic Assay: SK-OV-3 NTC and BRCA1-kd cells were exposed continuously to various concentrations of MK-2206, cisplatin, and olaparib. After 13 days, colonies were stained and clonogenic survival was determined. Data are means ± SD

In cytotoxicity assays, both BRCA2-proficient PEO4 and BRCA2-deficient PEO1 showed similar responses to MK-2206, with PEO4 demonstrating marginally higher susceptibility at all doses (Fig. 2a). The BRCA1-deficient SK-OV-3 BRCA1-kd cells showed greater susceptibility to MK-2206 at all doses as compared to BRCA1-proficient SK-OV-3 NTC cells (Fig. 2b). BRCA2-proficient PEO4 exhibited substantially decreased sensitivity to cisplatin across the dose range as compared to BRCA2-deficient PEO1 (Fig. 2a). Similarly, in clonogenic assays, SK-OV-3 BRCA1-kd cells were markedly more sensitive to cisplatin treatment than SK-OV-3 NTC cells (Fig. 2b). Both BRCA-wild-type cell lines showed minimal response to olaparib, with BRCA2-proficient PEO4 showing almost 100 % survival at even the highest dose of olaparib, while the BRCA mutants showed moderate sensitivity at all doses.

### Cisplatin and olaparib treatment induces AKT activation in BRCA2 mutant cells

**Fig. 3:**
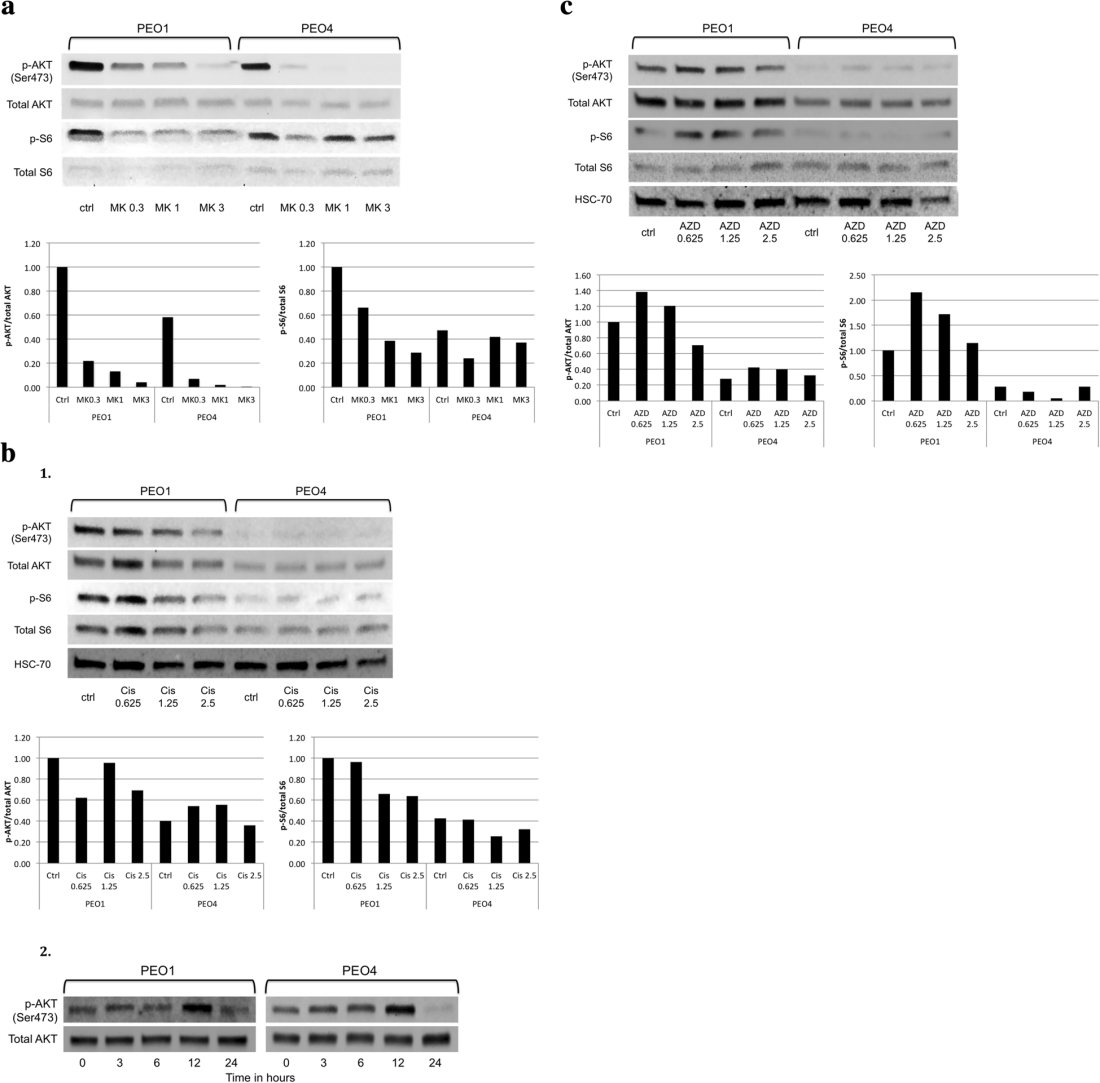
Effects of MK-2206, cisplatin, and olaparib on AKT activity in BRCA wild-type and mutant EOC cells. **a** PEO1 and PEO4 cells were treated with 0.3 μM, 1 μM, or 3 μM MK-2206 (MK) for 24 h. Band intensities quantified with ImageJ software. **b** 1. PEO1 and PEO4 cells were treated with 0.625 μM, 1.25 μM, or 2.5 μM cisplatin (Cis) for 24 h. 2. PEO1 and PEO4 cells were treated with 2.5 μM cisplatin (Cis) for 0, 3, 6, 12, and 24 h. **c** PEO1 and PEO4 cells were treated with 0.625 μM, 1.25 μM, or 2.5 μM olaparib (AZD) for 24 h. Total proteins were assessed for the levels of phosphorylated and total AKT and S6 by western blot analysis. HSC-70 protein levels were also used as a loading control. The ratios of phosphorylated protein to total protein relative to that of the control of PEO1 (set as 1) are shown in bar graphs

We then conducted western blot analysis to assess the impact of single agent treatments on AKT phosphorylation and activity. As expected, treatment of all cell lines with MK-2206 downregulated AKT and S6 phosphorylation in a dose-dependent manner in both PEO1 and PEO4 (Fig. 3a). Downstream phosphorylation of S6 was also decreased, although with a less precise dose–response relationship. At 24 h, cisplatin treatment resulted in lower levels of AKT and S6 phosphorylation than at baseline in the PEO1 BRCA2-deficient cells (Fig. 3b). AKT phosphorylation was largely unaltered by cisplatin treatment in the PEO4 BRCA2-proficient cells at 24 h. A similar dose-responsive decrease in p-S6 levels is apparent in the BRCA2-deficient but not in the BRCA2-proficient cells (Fig. 3b). However, when the experiment was repeated with PEO1 and PEO4 cell lysates collected at shorter post treatment time intervals, increased phosphorylation of AKT is observed in both PEO1 and PEO4 cells. Maximum activation of AKT is observed at 12 h post treatment, with subsequent depletion below baseline by the 24 h time point. Olaparib treatment induces AKT activation in the BRCA2 mutant PEO1. We also detected mild induction of AKT phosphorylation in the BRCA-proficient PEO4. S6 phosphorylation reflects the trend of AKT activation in the BRCA2 mutant PEO1 (Fig. 3c).

### MK-2206 sensitizes BRCA2 mutants to cisplatin and olaparib therapy

**Fig. 4:**
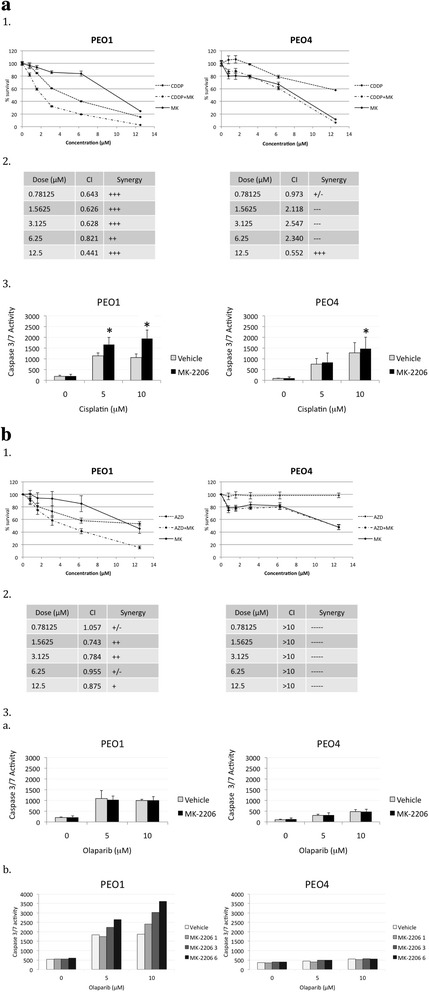
MK-2206 sensitizes BRCA2 mutants to cisplatin and olaparib therapy. **a** 1. PEO1 and PEO4 cells were treated with various concentrations of cisplatin, MK-2206, or both in combination at a constant 1:1 ratio. After 72 h, MTS solution was added and absorbance read at 490 nm after 4 h. Data are means ± SD. 2. CI values were calculated for the drug combination of cisplatin and MK-2206 at each concentration. 3. PEO1 and PEO4 cells were treated with increasing doses of cisplatin in combination with 1 μM MK-2206 and caspase 3/7 activity determined after 72 h. **b** 1. Assay repeated for the combination of olaparib and MK-2206. 2. CI values for olaparib and MK-2206 3. *a*. Caspase 3/7 activity assay for olaparib and MK-2206 (1 μM) . Asterisks, *p* < 0.05 (Student’s *t*-test) *b* Caspase 3/7 activity assay for olaparib and MK-2206 (1, 3, and 6 μM)

In cytotoxicity assays, MK-2206 demonstrated strong, dose-independent synergism with cisplatin treatment in the PEO1 BRCA2-deficient cells (Fig. 4a, 1). In contrast, MK-2206 antagonized the cytotoxic effects of cisplatin in BRCA2-proficient PEO4 at all but the highest dose (Fig. 4a, 2). In BRCA2-proficient PEO4 cells, the survival curve of the cisplatin and MK-2206 combination closely follows the survival curve of MK-2206 monotherapy. Similarly, co-treatment with MK-2206 selectively induced enhanced activation of caspase 3/7 in PEO1 cells treated with all doses of cisplatin while only resulting in slight but significant differential induction of apoptosis at the highest dose of cisplatin in PEO4 cells, likely in response to the overwhelming DNA damage at the highest doses of cisplatin (Fig. 4a, 3). The combination of olaparib and MK-2206 also resulted in mild to moderate, dose independent synergism in BRCA2-deficient PEO1 cells, while resulting in very strong antagonism at all doses in the BRCA2-proficient PEO4 line (Fig. 4b). Initial experiments demonstrated that co-treatment with MK-2206 failed to enhance caspase 3/7 activation in PEO1 cells treated with olaparib. We hypothesized that this discrepancy reflected that the chosen concentration of MK-2206 (1 μM) was too low to enhance apoptosis induced by olaparib (Fig. 4b, 3a). Increased concentrations of MK-2206 in combination with olaparib resulted in a clear dose responsive induction of caspase 3/7 activity in the BRCA2-deficient PEO1 cells while caspase 3/7 activity was unaffected in the BRCA2-proficient PEO4 even at maximal doses of olaparib and MK-2206 (Fig. 4b, 3b).

### MK-2206 prevents cisplatin- and olaparib-induced AKT phosphorylation

**Fig. 5:**
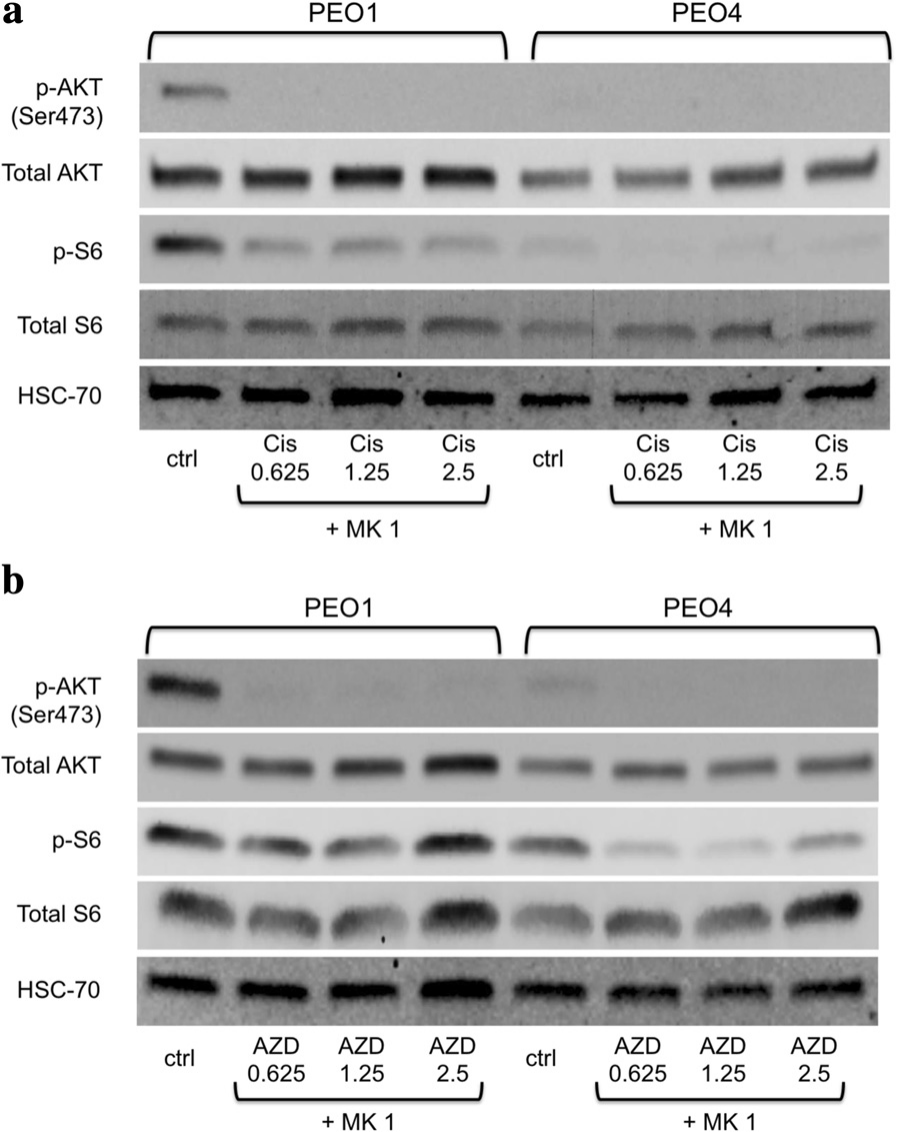
MK-2206 prevents cisplatin- and olaparib-induced AKT phosphorylation. **a** PEO1 and PEO4 cells were treated with 1 μM MK-2206 in combination with 0.625 μM, 1.25 μM, or 2.5 μM cisplatin for 24 h. Levels of phosphorylated and total AKT and S6, and HSC-70 proteins were assessed by western blot analysis. **b** PEO1 and PEO4 cells were treated with 1 μM MK-2206 in combination with 0.625 μM, 1.25 μM, or 2.5 μM olaparib for 24 h. Levels of phosphorylated and total AKT and S6, and HSC-70 proteins were assessed by western blot analysis

MK-2206 co-treatment repressed phosphorylation of AKT and S6 in the BRCA2 mutant PEO1 treated with cisplatin (Fig. 5a). AKT activity was reduced by MK-2206 treatment to undetectable levels at all doses of cisplatin despite robust levels of total AKT. *p*-S6 levels were also markedly reduced. In BRCA2-proficient PEO4, the low levels of *p*-AKT with cisplatin treatment were also obliterated by MK-2206. A similar trend was observed with the combination of olaparib and MK-2206 (Fig. 5b). MK-2206 inhibited the phosphorylation of AKT in the BRCA2 mutant PEO1 at all doses of olaparib, while *p*-S6 levels were reduced but still evident. In PEO4, the low level of *p*-AKT in olaparib-treated cells was reduced to undetectable levels by MK-2206 treatment. *p*-S6 levels were again reduced but still detectable.

### MK-2206 sensitizes BRCA2 mutants to combination therapy with cisplatin and olaparib

**Fig. 6:**
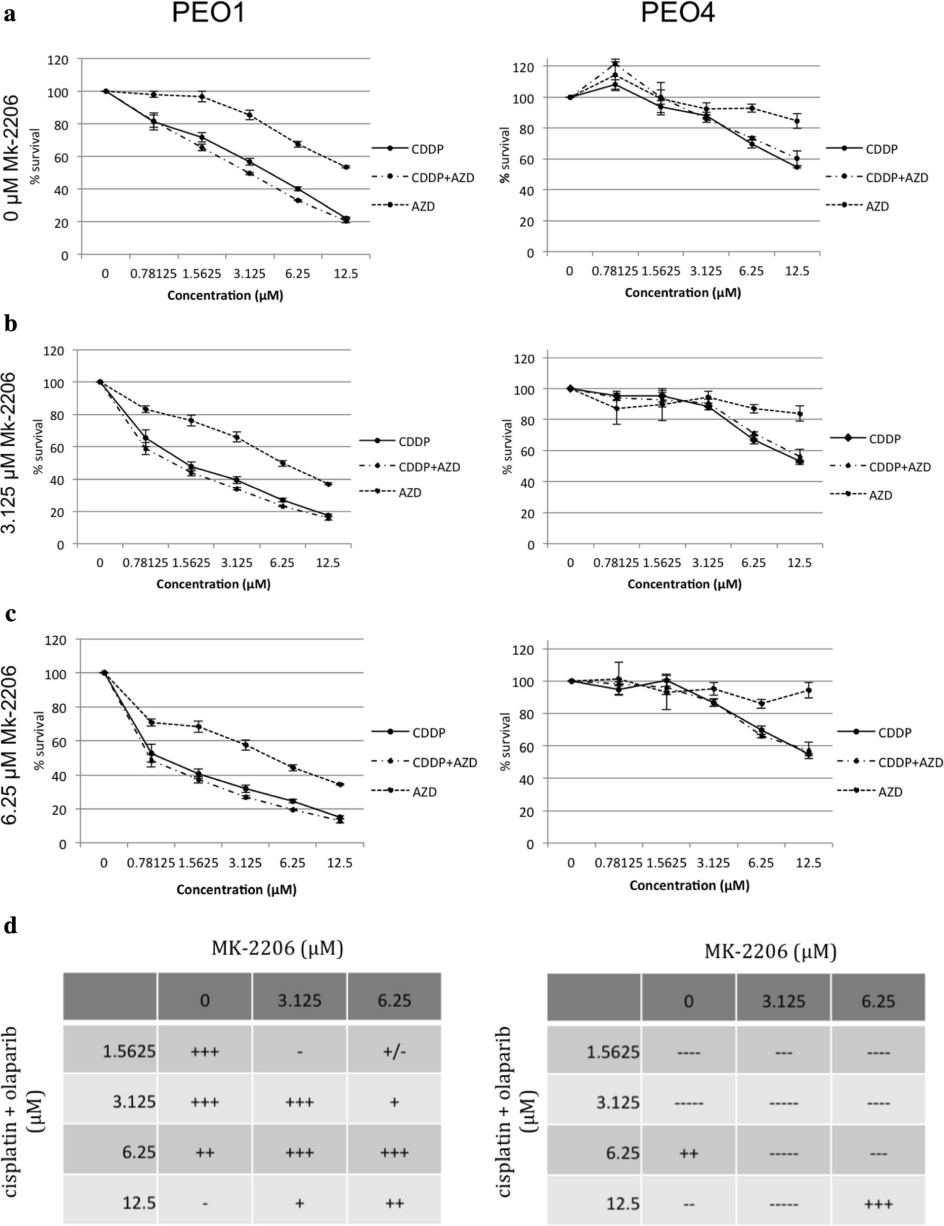
MK-2206 sensitizes BRCA2 mutants to combination therapy with cisplatin and olaparib. PEO1 and PEO4 cells were treated with (**a**) 0, (**b**) 3.125, or (**c**) 6.25 μM MK-2206 in combination with a fixed ratio (1:1) of cisplatin and olaparib. After 72 h, MTS solution was added and absorbance read at 490 nm after 4 h. Data are means ± SD. **d** CI values were calculated for the drug combinations of cisplatin and olaparib with MK-2206 at each concentration

We conducted cytotoxicity assays to assess the effects of cisplatin, olaparib, and MK-2206 in triple combination on PEO1 and PEO4. Cells were treated with a fixed 1:1 ratio of cisplatin and olaparib dosing and three fixed doses of MK-2206 (0, 3.125, and 6.25 μM). The combination of cisplatin and olaparib without MK-2206 showed moderate synergism at lower doses but a trend of diminishing synergism toward higher doses in BRCA2-deficient PEO1, while demonstrating antagonism at most doses in BRCA2-proficient PEO4 (Fig. 6d). The addition of MK-2206 showed mild to moderate synergism of the triple combination at the higher doses in PEO1 and moderate to strong antagonism at almost all tested doses in PEO4.

## Discussion

Chemotherapeutic sensitivity and resistance is a key factor in the treatment of EOC. AKT, which is involved in pro-survival and anti-apoptotic cell signaling pathways, has been implicated in chemotherapeutic resistance and has been identified as a potential target for chemotherapeutic strategies. In addition, novel chemotherapeutic strategies attempt to exploit the underlying weaknesses of the cancer cells themselves. BRCA1 and BRCA2 mutations in EOC cell lines result in compromised HR repair, the primary cellular mechanism for the repair of DSBs.

Here, we elucidate the relationship between BRCA1/2 deficiency and the AKT survival pathway, and propose a targeted chemotherapeutic strategy for BRCA1/2 mutant EOC. This study suggests that AKT activity is upregulated in BRCA1 and BRCA2 mutant EOC, and that the inhibition of AKT phosphorylation by the allosteric inhibitor MK-2206 sensitizes BRCA mutants to apoptosis induced by cisplatin and olaparib. Given the ability of AKT to override apoptotic signaling, we propose that the baseline increased AKT activity in BRCA mutant cells reflects not only the lack of the repressive function of BRCA but also enhancement of a primary survival mechanism in the presence of faulty DNA repair. Therefore, removing a fundamental compensatory mechanism renders BRCA mutant cells exquisitely sensitive to additional DNA damage caused by platinum drugs and PARP inhibitors.

First, western blot analysis suggests higher levels of AKT activation in both BRCA1 and BRCA2 mutant cell lines at baseline prior to any drug treatment. Both SKOV3 BRCA1-kd and BRCA2 mutant PEO1 showed substantial phosphorylation at the Ser473 site and SKOV3 BRCA1-kd also showed a low level of increased phosphorylation at the Thr308 site. As mentioned previously, our experiments did not demonstrate AKT phosphorylation at the Thr308 site in any of the PEO1/4 cells. Whether this discrepancy reflects a true difference in the activation of the two phosphorylation sites is unclear. While the differential effects of phosphorylation at the two sites has not been fully characterized and are a subject of ongoing investigation, defects in Ser473 phosphorylation have been shown to affect only a subset of the total targets of AKT function. For example, S6-kinase phosphorylation (the kinase that when activated phosphorylates S6), notably, was not affected in vivo by lack of Ser473 phosphorylation, and AKT that is phosphorylated at only the Thr308 site showed continued partial activity [41]. Investigation into the exact mechanisms of AKT to S6 signaling is ongoing. Treatment with increasing doses of MK-2206 resulted in a clear dose-responsive downregulation of AKT phosphorylation in both BRCA-proficient and deficient cells. Although we observed a corresponding downregulation of S6 phosphorylation, there is not the same dose response. The inexact correlation between AKT phosphorylation and S6 phosphorylation may be attributable to the independence of S6 from the Ser473 site discussed previously. However, treatment with olaparib appears to induce phosphorylation of both AKT and S6 in BRCA2 mutant cells while still showing no activation at the Thr308 site. The fact that S6 is activated in sequence with Ser473 suggests that their function is either more closely linked than was previously indicated, or that this western blot assay does not accurately reflect levels of Thr308 phosphorylation.

In contrast to olaparib treatment in PEO1 cells, initial studies with treatment with cisplatin resulted in decreased levels of phosphorylated AKT at 24 h. However, previous work in HCT-116 human colon carcinoma cells revealed maximum induction of AKT phosphorylation at 6 h post treatment with cisplatin. At 24 h post treatment, *p*-AKT levels were found to be below baseline after the initial intense activation of AKT in response to the devastating DNA damage resulting from cisplatin treatment [42]. In this context, we hypothesized that the apparent downregulation of AKT activation in response to cisplatin activation in fact represented depletion of *p*-AKT after maximum activation earlier in the time course. Subsequent experiments revealed maximum levels of *p*-AKT at 12 h with subsequent depletion by the 24 h mark in both PEO1 and PE04 cell lines. The apparent inverse dose response of AKT activation at 24 h with increasing dose of cisplatin may therefore represent increased depletion of *p*-AKT after a progressively more intense, dose-proportionate response to greater DNA insult. The uniformity of the phosphorylation level of AKT in PEO4 across all doses of cisplatin at 24 h,may be reflective of the cisplatin resistance inherent in the BRCA-proficient line. As PEO4 has intact HR repair and is better equipped to manage platinum–induced DNA damage, it is less reliant on AKT activation for apoptotic override, and the dose response relationship is not as precise.

Consistent with published data, both of the BRCA-deficient cell lines demonstrated better apoptotic response to cisplatin and olaparib. As we have established that the BRCA2 mutant cells have higher AKT activity at baseline and then further increase AKT activity in response to DNA damage, it is unlikely, despite the known connection between AKT activation and platinum resistance in other models, that the difference in platinum resistance between the BRCA-proficient and -deficient cells is fully mediated by increased AKT activation in the BRCA-proficient cells. The increased sensitivity to olaparib is consistent with the literature and attributable to the synthetic lethality of PARP inhibition in the setting of defective HR repair in BRCA2-deficient cells [43, 44].

The combination of cisplatin with MK-2206 in the BRCA2 mutant showed moderate synergism at all doses. As cisplatin treatment alone appears to upregulate AKT phosphorylation in the BRCA mutant line, it is likely that AKT activation is a key mechanism for survival in the face of platinum-induced DNA damage. When this mechanism is impeded, the BRCA-deficient cells are unable to mount a response against the cisplatin-induced apoptosis. In contrast, AKT inhibition with MK-2206 antagonizes the action of cisplatin in PEO4. It is unlikely that the cisplatin resistance in PEO4 is completely attributable to upregulated AKT activity, and so the inhibition of AKT has little impact on the cytotoxic effects of cisplatin. The strong synergism seen at the highest dose of the cisplatin and MK-2206 in combination in PEO4 is likely mathematically attributable to the high overall kill rate at the highest drug doses.

MK-2206 exhibited mild to moderate synergism with olaparib in the BRCA2 mutant at most doses. The combination of olaparib and MK-2206 in the BRCA2 mutant is unique in that it may create two separate mechanisms of synthetic lethality. As described above, the BRCA mutant may be reliant on AKT activity at baseline, and thus is particularly susceptible to AKT inhibition. Olaparib, as a PARP inhibitor, compromises NER and compounds the BRCA2 mutant’s underlying defect in HR repair. In contrast, as the BRCA2-proficient PEO4 cell line was predictably unresponsive to olaparib at all doses, the overall survival of the cells exposed to the combination is essentially the same as treating them with MK-2206 alone. The less dramatic response of the BRCA mutant cells to the olaparib and MK-2206 combination as opposed to cisplatin and MK-2206 may be attributable to less intrusive DNA damage and cytotoxicity caused by the tested dose range of olaparib, which requires HR for repair, as compared to cisplatin, which requires both HR and NER. In BRCA mutant cells, olaparib induces the accumulation of DSBs, as existing single-strand breaks stall the replication fork and are converted to DSBs in the absence of functional PARP [45]. In contrast, the DNA adducts formed by cisplatin cross-links induce the formation of both double- and single-strand breaks and activate a broad range of proapoptotic pathways including ATR, p53, p73, and MAPK [46].

While olaparib and MK-2206 each have some individual cytotoxic effect, they are likely to be most effective in triple combination with cisplatin or another DNA-damaging agent. Our studies of the triple combination demonstrate mild to moderate synergism of the triple combination of cisplatin, olaparib, and MK-2206 in the BRCA2-deficient PEO1. In contrast, the triple combination showed strong antagonism at almost all tested doses in BRCA2-proficient PEO4. It is important to note, however, that the combination of cisplatin and olaparib alone, without MK-2206, showed synergism at lower doses, and there did not appear to be a dramatic increase in synergism with the addition of MK-2206. Here we may face the limitations of the Chou-Talalay method. Due to limitations in the practicality of multiple drug dosing in the 96-well plates for cytotoxic assay, cisplatin and olaparib were dosed in a 1:1 fixed ratio, while MK-2206 was added in two fixed levels. We speculate that the mathematics of the Chou-Talalay method favor fixed ratio drug combinations [47], and therefore may be less sensitive to added synergism due to MK-2206 in this experiment. Additionally, treating cells in a continuous manner for 72 h with cisplatin and olaparib rather than conducting washout of the drug after a short interval may induce DNA damage too profound to be dramatically affected by further inhibition of AKT. Further examination with alternative experimental design is necessary for additional confirmation of synergistic phenomena with the triple drug combination treatment.

A limitation of this study is the use of monolayer cell culture as our primary experimental modality, given the discrepancy between condition in monolayer cell cultures and the in-vivo environment they are intended to model. To this end, further work in both spheroid cell models and in-vivo mouse models is currently ongoing in our laboratory.

## Conclusion

In summary, by targeting the baseline reliance of the BRCA mutant cells on AKT activity for survival, we created a synthetic lethal combination with MK-2206. The BRCA mutant, having already exhausted a potent survival mechanism in the face of compromised HR repair, is unable to withstand the DNA damage from cisplatin or olaparib. AKT inhibition by MK-2206 produces a unique synthetic lethality and will potentially sensitize BRCA mutants to DNA-damaging and PARP -inhibitor therapy for ovarian cancer.

## Abbreviations

AZD-2281, Olaparib; BRCA1-kd, BRCA1-knockdown; cis/CDDP, cisplatin; EOC, Epithelial ovarian cancer; HR, Homologous recombination; NTC, Non-targeted control; PARP, Poly (ADP-ribose) polymerase
